# Computational fluid dynamics dataset of secondary air modification in a circulating fluidized bed boiler

**DOI:** 10.1016/j.dib.2023.109931

**Published:** 2023-12-10

**Authors:** Nurdin Hasananto Teguh, Lilis Yuliati, Eko Siswanto, Djarot B. Darmadi

**Affiliations:** Department of Mechanical Engineering, Faculty of Engineering, Brawijaya University, Indonesia

**Keywords:** Coal combustion in CFB boiler, Secondary air inlet, Air penetration, ANSYS-Fluent

## Abstract

Computer simulation has been proven to provide a good understanding of engineering phenomenon. This work presents numerical simulation results on secondary air jet penetration into a dense phase of a three-dimensional fluidized bed at a commercial scale. Initial model as a reference and four modified models which are called as case A, B, C, and D were created by modifying the angle of secondary air. Evaluation of combustion process is based on mass fraction distribution of H_2_O and CO_2_ at center line of the furnace. Generally, modified geometry improves the performance of furnace compared to reference. We also present data of total energy and temperature to get a comprehensive insight of the furnace performance. The simulation results can be used as a consideration to improve the efficiency of steam power plants by adjusting the direction of secondary air flow.

Specifications Table

**Table:** 

Subject:	Mechanical Engineering
Specific subject area:	Technical data generated from computational modelling of coal combustion in a Circulating Fluidized Bed Boiler
Data format:	Raw
Type of data:	Images, Charts, Graphs, Figures, Tables
Data collection:	Input parameters for combustion modelling in the CFB boiler were obtained from operational data of the Anggrek steam power plant which is located at Gorontalo, Indonesia. These data are recorded in the central control room of the steam power plant, including mass flow and composition of fuel (coal), mass flow of primary and secondary air as well as furnace geometry of the CFB boiler. Boiler combustion chamber as the computational domain was created using CAD software. The model was exported to ANSYS - Fluent, and grid generation was conducted using tetrahedral mesh type. For each model, a constant parameter was set in order to determine the effect of modified geometry and hydrodynamics that occurred inside the boiler combustion chamber. Several parameters obtained from the numerical simulation results are velocity distribution, penetration depth, flue gas temperature, mass fraction of fuel, oxidizer and combustion product. Several position in geometrical model were created and used as studied parameters such as an additional plane parallel with secondary air inlet at lower and upper sides. Those plane was used as scale to determine the depth of penetration. The temperature measurement was taken from outlet section. For determining the value temperature outlet/plane *y−*2 m/plane *y−*3.5 meter, we used “function area”, then selected location (plane1 *y−*2 m/plane *y−*3.5 m/outlet), then we filtered parameter (temperature) then recorded the value. For capturing picture, we used “save as picture”, then selected PNG/JPEG For determining the depth of penetration, we create line that in line with directional from inlet section. then we used ratio of width and length of cross section plane (3312 and 4056 mm) to normalize sizes. We used reference or based model that was created based on drawing document, then value of parameter was collected from actual operational data. Then, case A, B, C, D were gained by modifying base/reference 3D model. Center line from furnace section were defined then measured by chart function and extracted into csv file. The combination from several parameters (fuel, $CO_{2},\;O_{2},\;H_{2}O$ mass fraction, total energy, velocity and temperature) is visualized into graphical form and stored in Mendeley data.
Data source location:	Anggrek steam power plant at Gorontalo, North Sulawesi, Indonesia.
Data accessibility:	Repository name: Mendeley Data Data identification number: 10.17632/m362fgpjd7.2 Direct URL to data: Dataset of CFD simulation modified air angle of CFB Boiler - Mendeley Data

## 1 Value of the Data

• There is a limited amount data available on the modified industrial scale boiler model and the replication was done by numerical investigation. This can be beneficial for power plant engineer to digitally optimized their combustion chamber. The amount of detail data available on modified industrial scale boilers is limited, then the results of this modelling are a solution to these unavailability data. It can be useful for power plant engineers to optimize their combustion chambers using numerical methods.
• The data is useful for understanding and studying of occurred phenomenon in commercial scale circulating fluidized bed boiler. It can be used as a basis for improving and optimizing the combustion process in CFB boilers
• The data can be beneficial to fluidized bed researcher in academia to enhance their knowledge about distribution of $CO_{2},\;O_{2},\;H_{2}O$ towards elevation.
• The data can be useful for crucial insight planning such modification of modified secondary air angle or other redesign boiler geometry. The data is important for determining the inlet angle or secondary air flow direction that produces maximum boiler performance.
• The data temperature distribution can be elaborated more to predict the future malfunction/shortage due to erosion or high thermal stress.
• The data of total energy and temperature distribution might become prediction of actual heat transfer inside the combustion chamber.

## 2 Data Description

This paper presents a set of data associated with numerical simulation of modified secondary air angle of CFB boiler. The dynamics of air and fuel flow, combustion process and temperature distribution are simulated using ANSYS Fluent software. We have obtained data from simulation cases A, B, C, D, and reference. Reference condition is the modelling of combustion process in CFB boiler appropriate to actual operational condition, while the other conditions are modelling of combustion process in CFB boiler with variation in secondary air flow direction. The data then saved and stored in the Mendeley data. There are three folders and one excel raw data file. The first folder is “ANSYS Files” which contained resulted files from ANSYS Fluent simulation for five cases. The second folder is “CSV Files” which consist of five subfolder following five simulated cases. The last is the “Pictures” which collected all resulted pictures included image shown in this article. A complete folders structure can be seen in Tables 1 and 2.

**Table 1:** Folder structure from Mendeley data link.

File path	File description
ANSYS Files	Numerical result of case A, B, C, D, and reference. This files contained ANSYS format file.
CSV Files	Files of comma separated value from that extracted from ANSYS files.
Pictures	Files that contained images (upper, lower, left, right and middle) from case A, B, C, D, and reference model that extracted from CFD-Post.
Combined raw data	Excel file that contained combination of raw data of Fuel, CO_2_, H_2_O, O_2_ mass fraction, Total energy, Velocity, and Temperature towards elevation.

**Table 2:** Folder structure from “CSV files”.

File path	File description
Reference model	Numerical results of combustion process modelling of Reference case, consisted of Fuel, CO_2_, H_2_O, O_2_ mass fraction, Total energy, Velocity, and Temperature toward elevation from initial condition (base design).
Case A	Numerical results of combustion process modelling of Case A, consisted of Fuel, CO_2_, H_2_O, O_2_ mass fraction, Total energy, Velocity, and Temperature toward elevation at combustion chamber center line.
Case B	Numerical results of combustion process modelling of Case B, consisted of Fuel, CO_2_, H_2_O, O_2_ mass fraction, Total energy, Velocity, and Temperature toward elevation at combustion chamber center line.
Case C	Numerical results of combustion process modelling of Case C, consisted of Fuel, CO_2_, H_2_O, O_2_ mass fraction, Total energy, Velocity, and Temperature toward elevation at combustion chamber center line.
Case D	Numerical results of combustion process modelling of Case D, consisted of Fuel, CO_2_, H_2_O, O_2_ mass fraction, Total energy, Velocity, and Temperature toward elevation at combustion chamber center line.

**Fig. 1:**
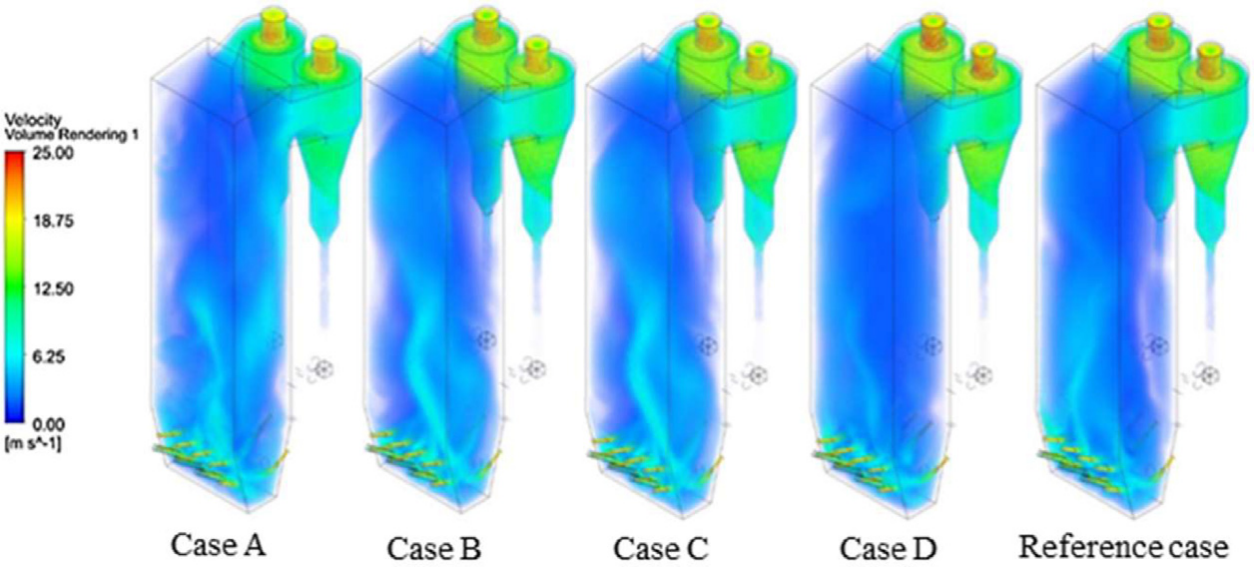
Isometric volume rendering of velocity distribution.

**Fig. 2:**
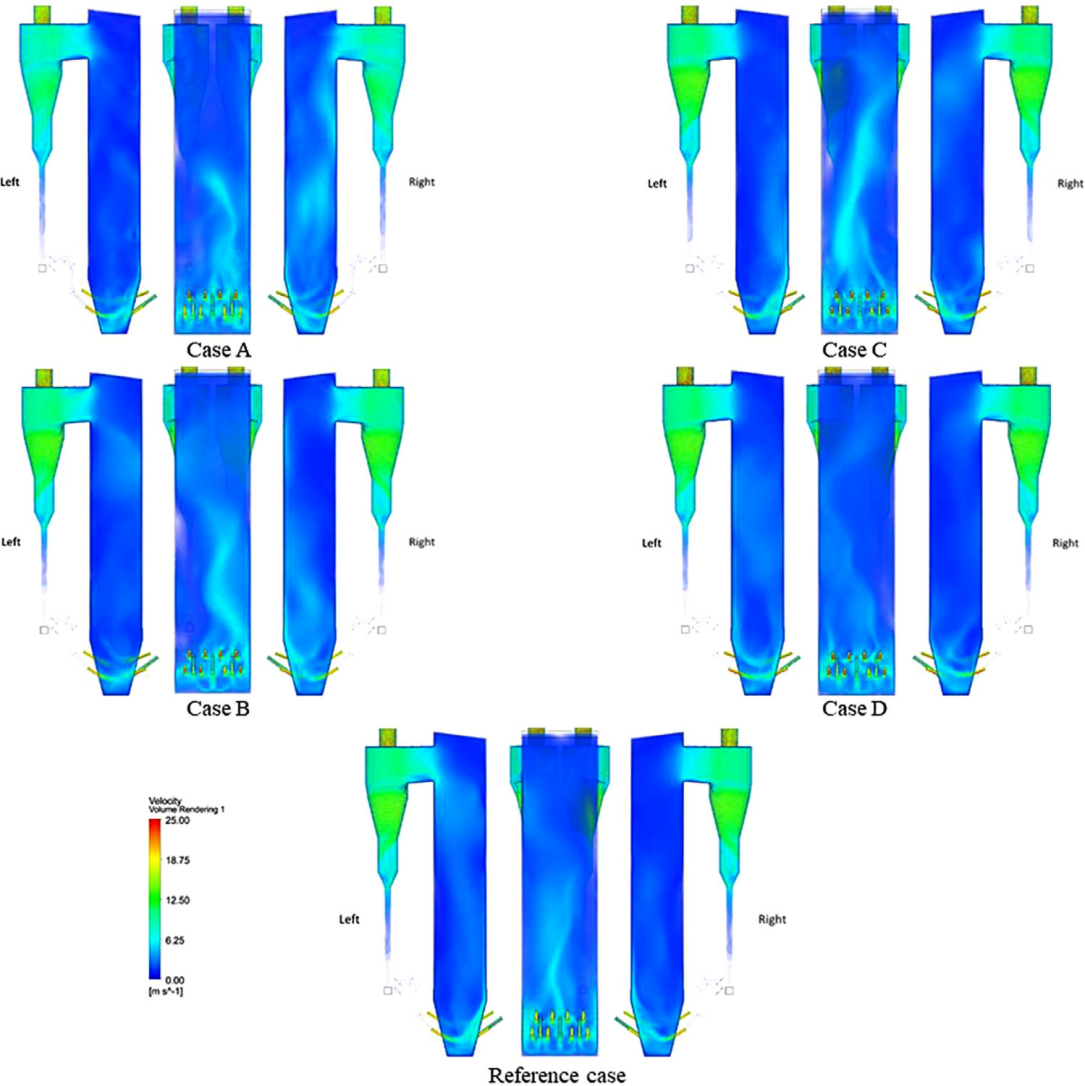
Volume rendering of the velocity distribution at different three planes inside the combustion chamber.

**Fig. 3:**
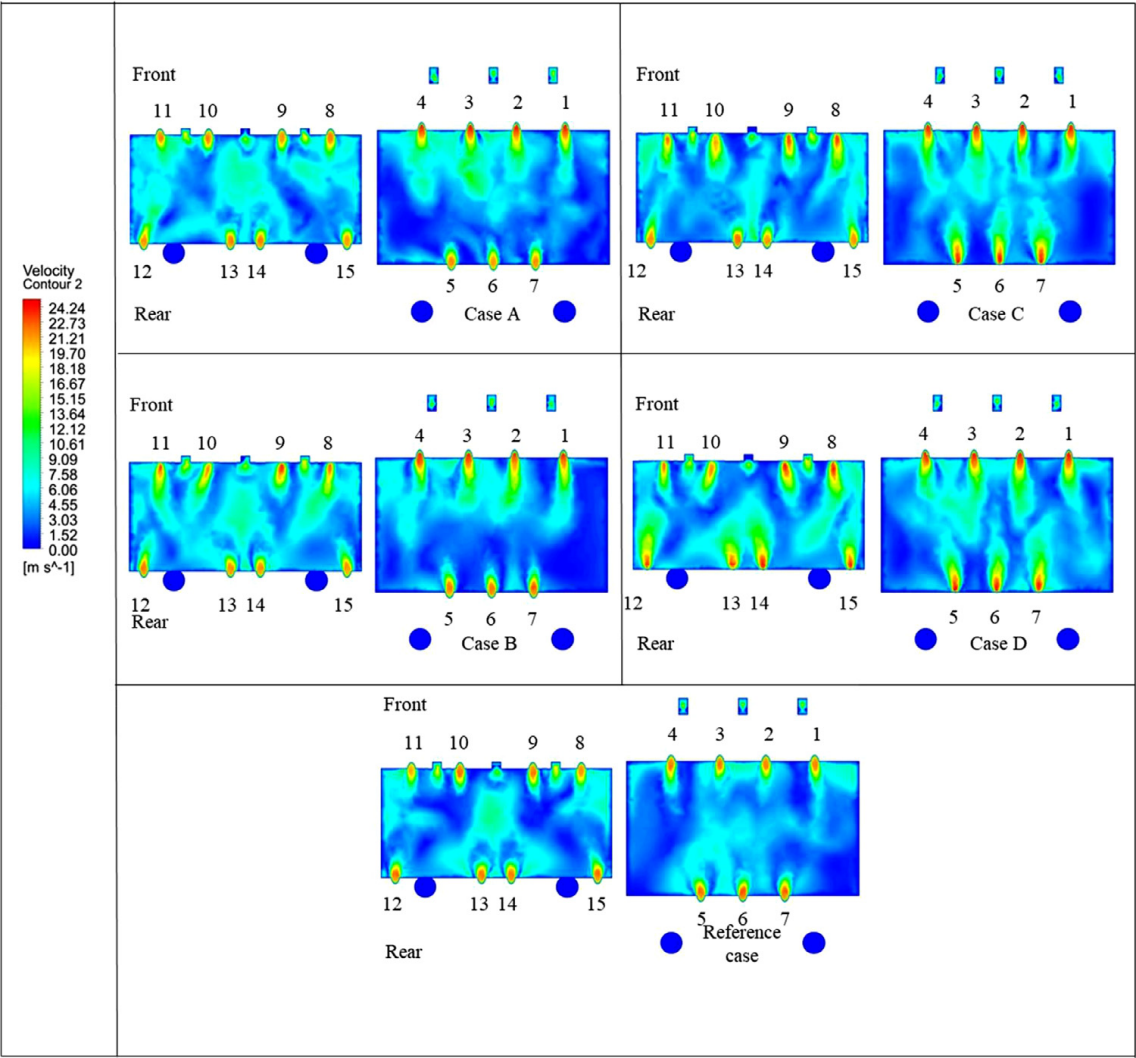
Velocity contour at elevation Y = 2 m and Y = 3 m.

**Fig. 4:**
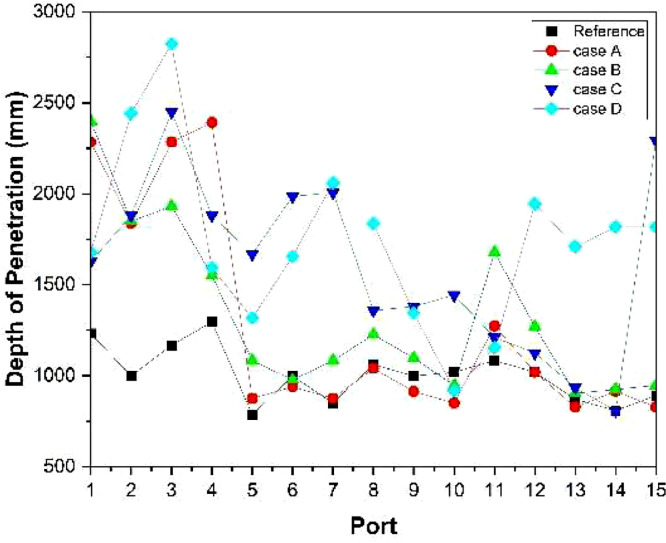
Depth penetration compared for each case.

**Fig. 5:**
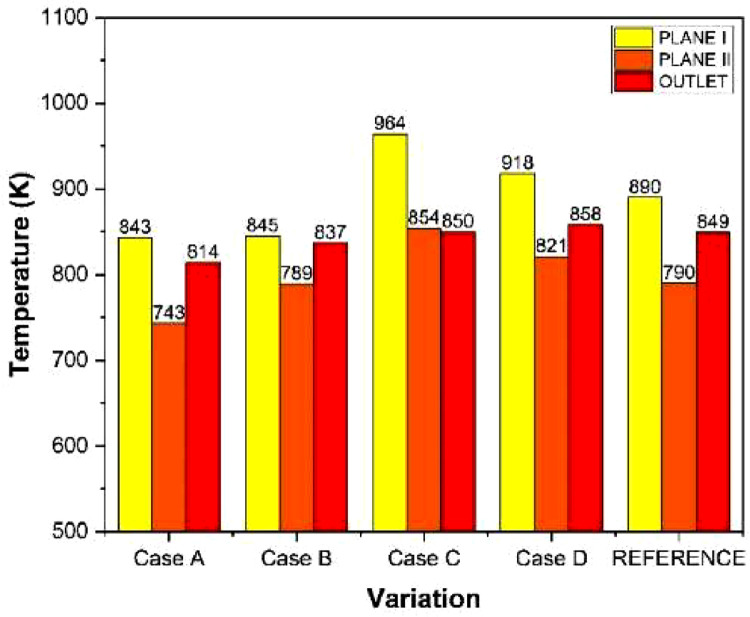
Average temperature of plane I, II and outlet.

**Fig. 6:**
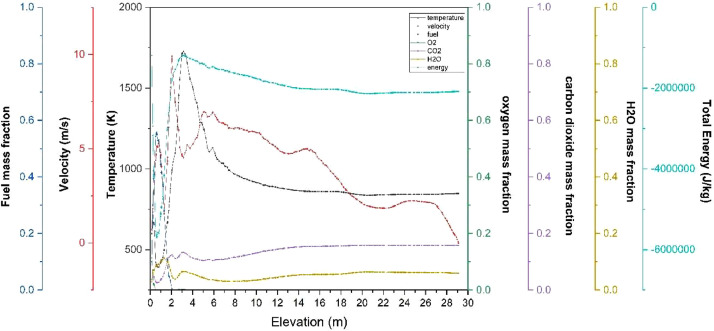
Combustion parameters measured along center line toward elevation (Reference case).

**Fig. 7:**
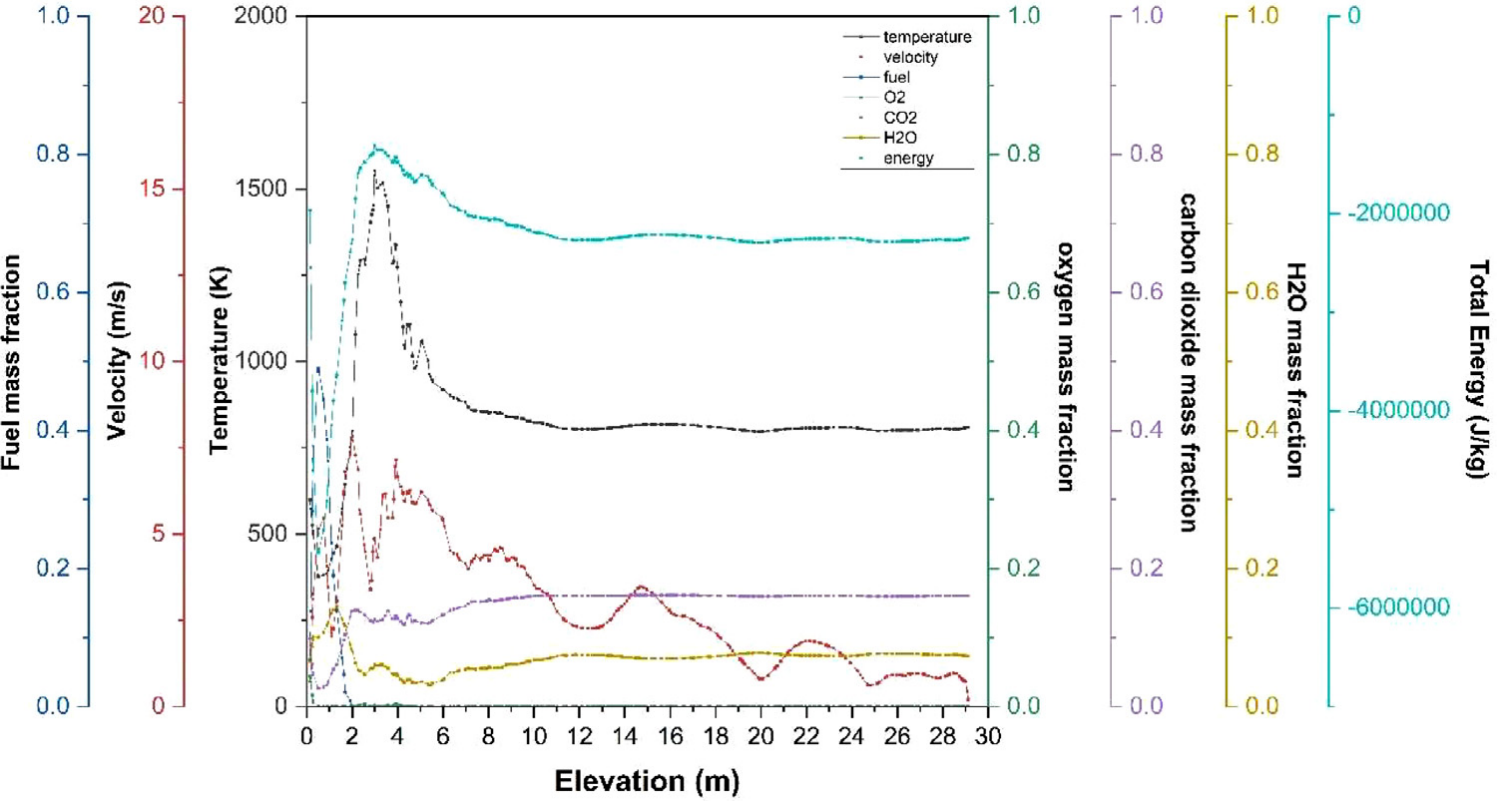
Combustion parameters measured along center line toward elevation (case A).

**Fig. 8:**
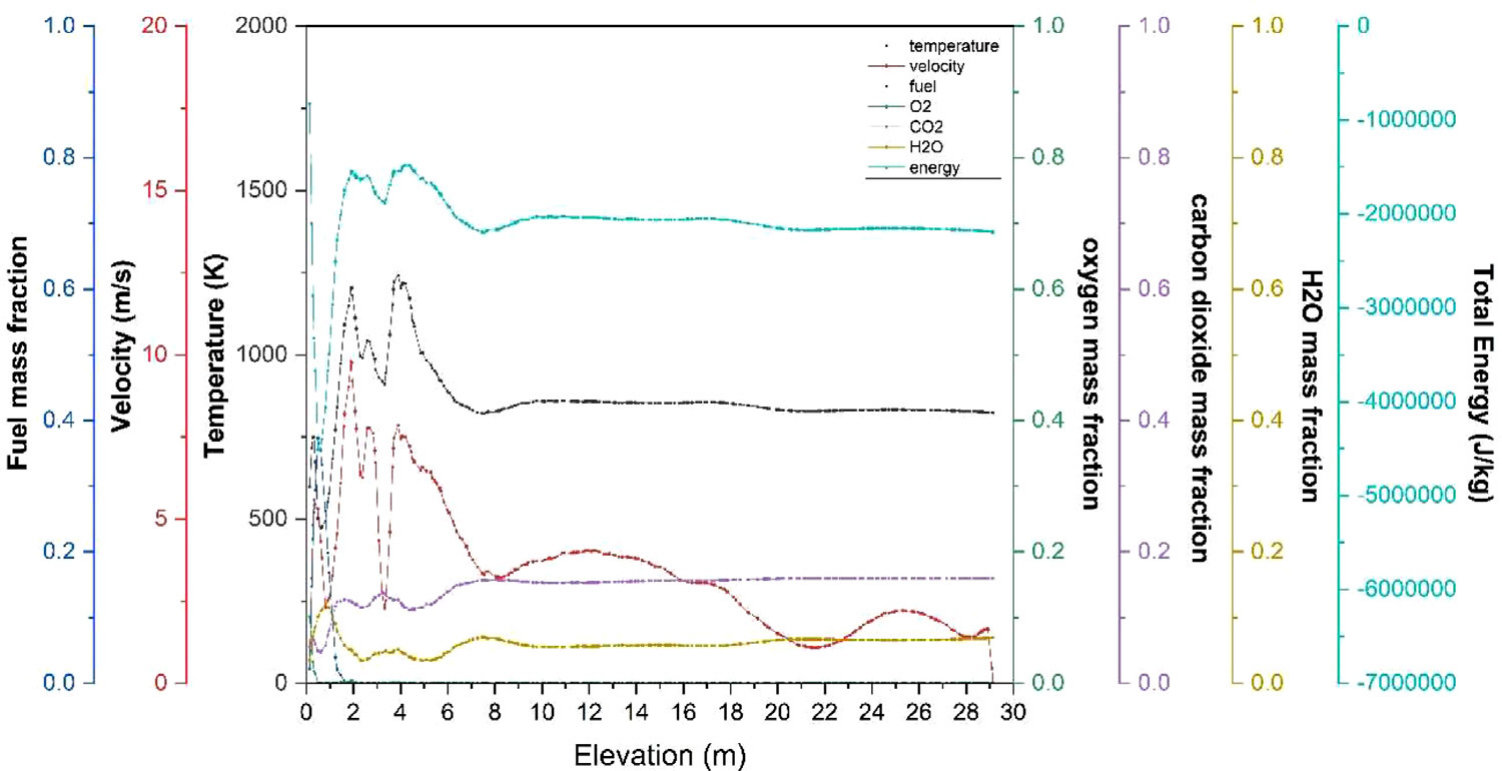
Combustion parameters measured along center line toward elevation (case B).

**Fig. 9:**
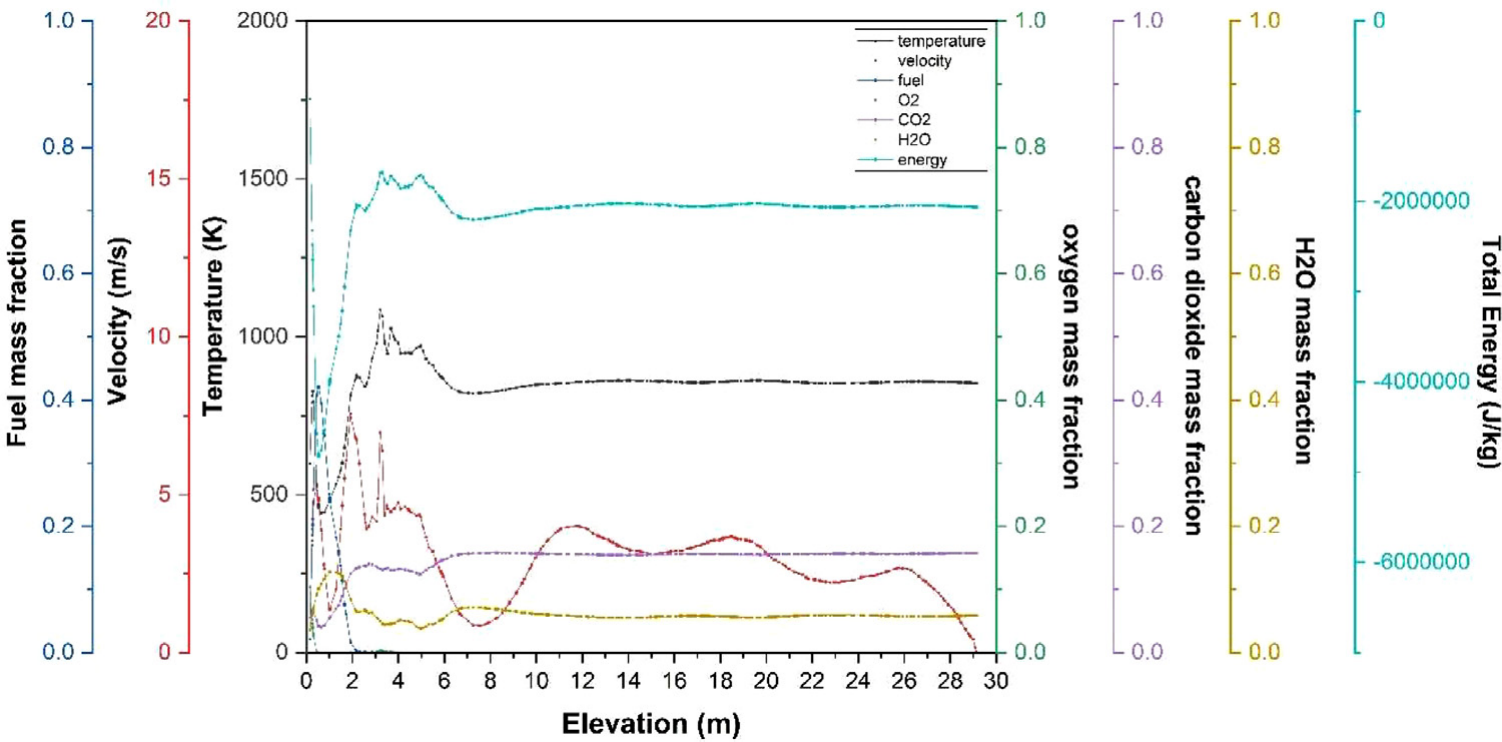
Combustion parameters measured along center line towards elevation (case C).

**Fig. 10:**
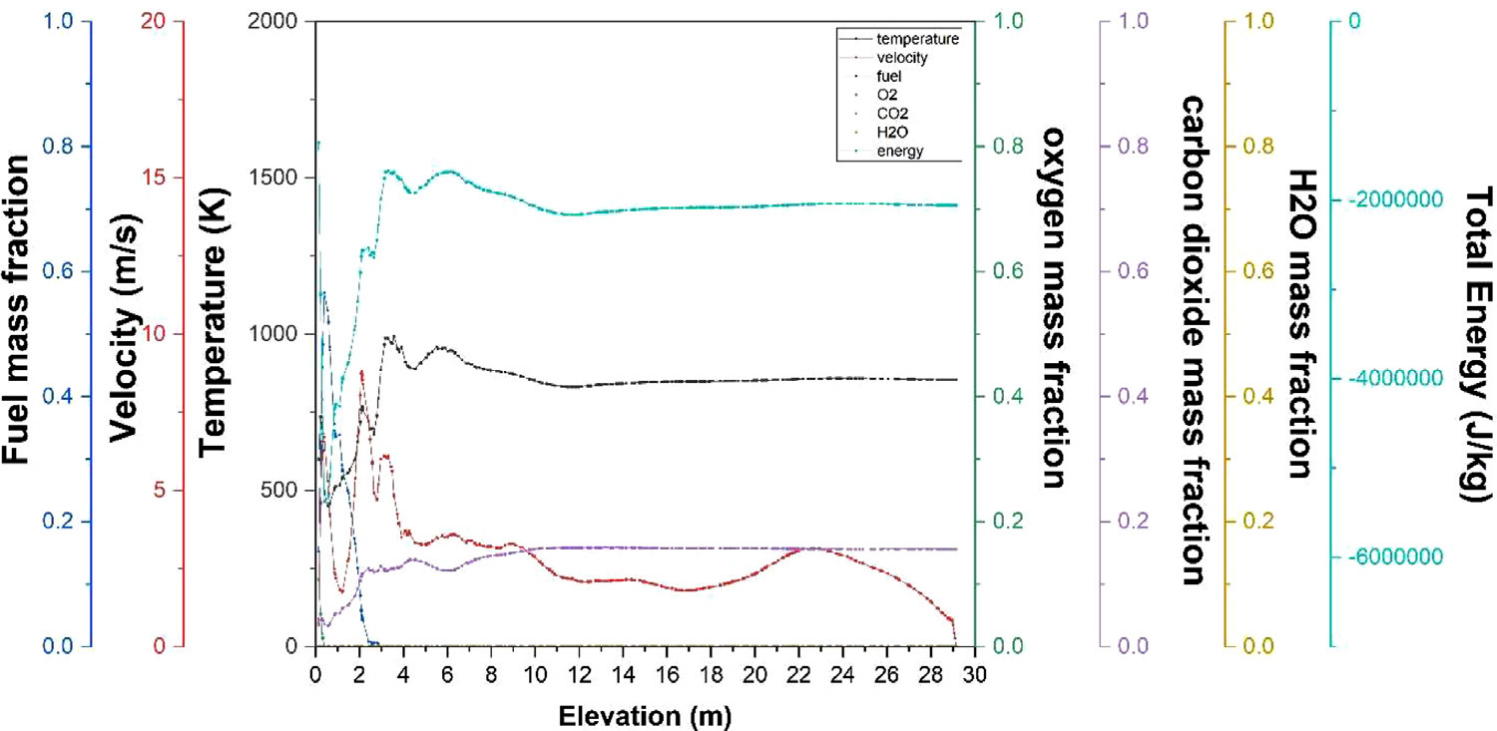
Combustion parameters measured along center line toward elevation (case D).

The numerical result is discussed below in terms of figures and graphs. Fig. 1 shows the isometric contour of velocity distribution in case A, case B, case C, case D and reference case. Fig. 2 displays the contour of the velocity distribution at different three planes inside the combustion chamber for case A, case B, case C, case D and reference case. The three planes consist of the *y*–*z* plane parallel to the axis of the left cyclone, the *x*–*y* plane through the center line of combustion chamber (as shown in Fig. 16), and the *y*–*z* plane parallel to the axis of the right cyclone. This figure is intended to provide a better point of view compared to isometric contour display. Fig. 3 shows the velocity contour in case A, case B, case C, case D and reference case where the left side describe top view of boiler with elevation 2 m (plane I), whilst the right side describe top view of boiler with elevation 3.5 m (plane II). Fig. 4 shows simulation results of jet penetration under different orientation angle with constant velocity magnitude of each air inlet. The penetrations were determined from velocity component normalized to the real furnace length in *z*direction. Fig. 5 shows temperature of plane I, plane II and outlet section, visualized into bar chart. Since furnace is a vital equipment, we further created more detail assessment was made by evaluating several output parameters from CFD simulation. Fig. 6, Fig. 7, Fig. 8, Fig. 9, Fig. 10 show the fuel, $CO_{2},\;H_{2}O,\;O_{2}$ mass fraction, total energy, velocity, and temperature measured along the center line of the combustion chamber for Reference case, Case A, Case B, Case C, and Case D respectively. The center line of the combustion chamber is shown in Fig. 16. The raw data for Fig. 6, Fig. 7, Fig. 8, Fig. 9, Fig. 10 can be seen in “CSV Files” folder which consist of five (5) sub folders, i.e. Reference case, Case A, Case B, Case C, and Case D folders. Each sub folder for all case contains seven (7) files containing data of the fuel, $CO_{2},\;H_{2}O,\;O_{2}$ mass fraction, total energy, velocity, and temperature respectively in CSV format (Table 3).

**Table 3:** Coal composition.

Parameter	(Mass fraction)
Volatile matter	0.5
Fixed carbon	0.3
Ash	0.1
Moisture	0.1
C	0.85 (DAF)
H	0.1 (DAF)
O	0.04 (DAF)
N	0.01 (DAF)
S	0 (DAF)

## 3 Experimental Design, Materials and Methods

This work was developed in the facilities of the Mechanical Engineering Laboratories at the University of Brawijaya. The model created in Autodesk Fusion was simulated by applying computational fluid dynamics using commercial code ANSYS - Fluent. The design and simulations were carried out on desktop computer with an intel i7 13xx, 32 GB of RAM and NVidia RTX 3060 graphics card.

### 3.1 CAD modelling using Autodesk Fusion software

**Fig. 11:**
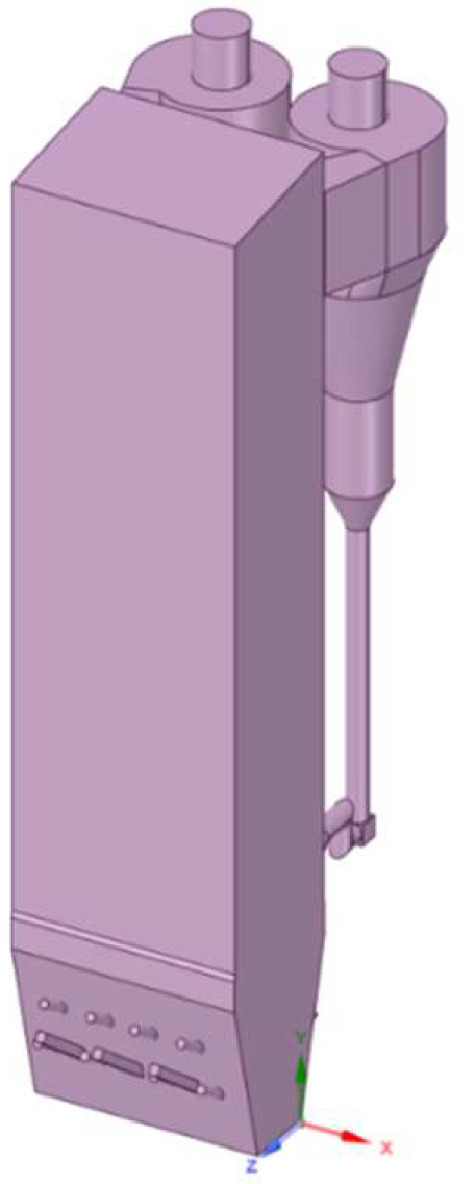
Model of combustion chamber in circulating fluidized bed boiler.

**Fig. 12:**
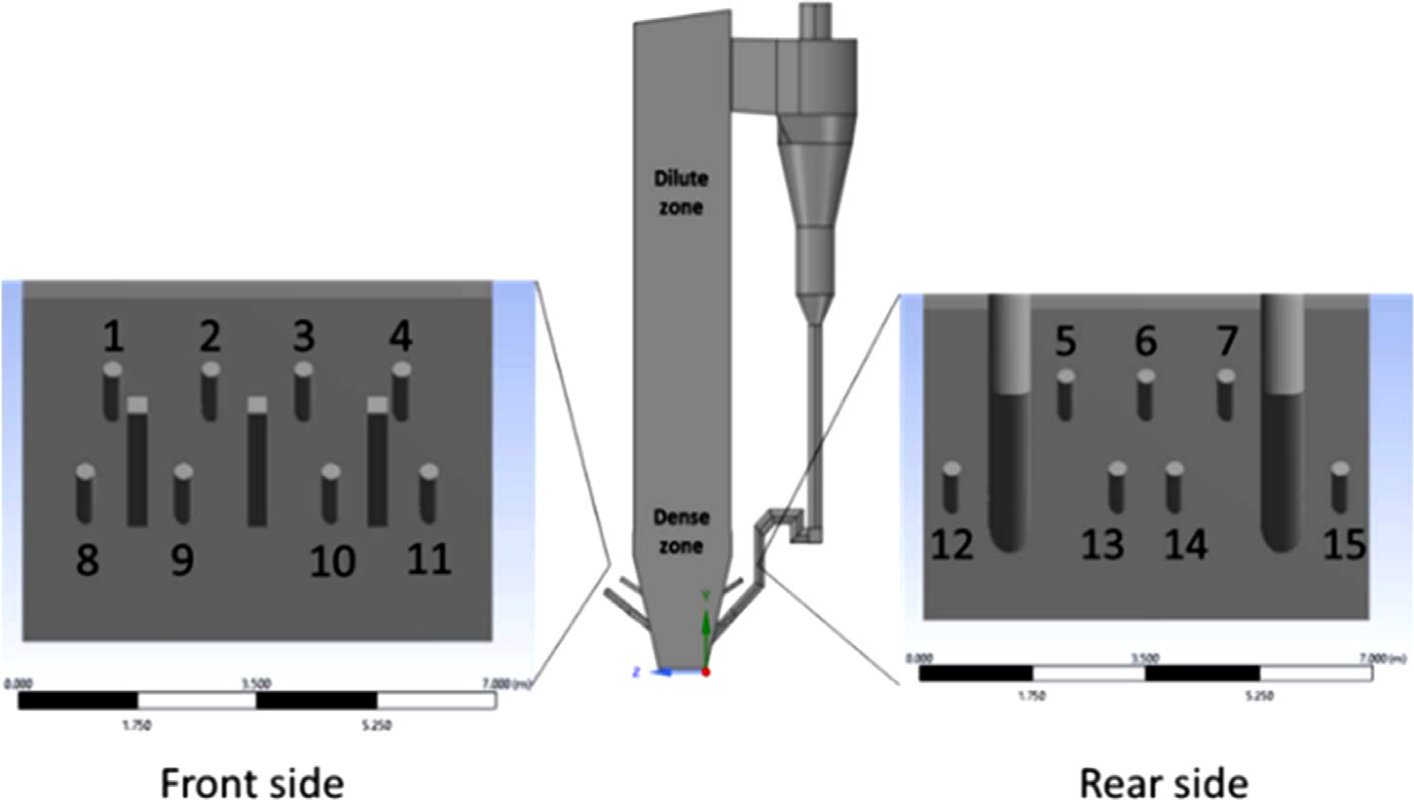
Configuration of secondary air inlet on CFB model (channel no 1–15).

The design of the combustion chamber model is based on actual size of commercial scale boiler of Anggrek steam power plant located in North Gorontalo Indonesia. Fig. 12 shows the model of circulating fluidized bed boiler, this model includes main combustion chamber, primary air inlet at the bottom, secondary air inlet nozzle, coal inlet port, two cyclones and return leg. There are 15 secondary air inlet consisting of the upper front side group (1–4), the lower front side group (8 -9), the upper rear side group (5–7) and the lower rear side group (12–15), as indicated in Fig. 12. We have simplified the primary air inlet as rectangular plane at the bottom of the furnace with uniform primary air flow and did not consider the nozzle from return leg for reducing computational cost. It has dimensions of 6720 mm in length, 4560 in width, and 29740 mm in height representing commercial scale of boiler. Base model/reference case is presented in Fig. 11, showing the modelling of coal combustion in a CFB boiler according to the actual boiler geometry and input parameters according to operational conditions. Then, we present modelling of coal combustion by varying the direction of the secondary air inlet flow, as shown in Fig. 15.

### 3.2 Mesh independence test

**Fig. 13:**
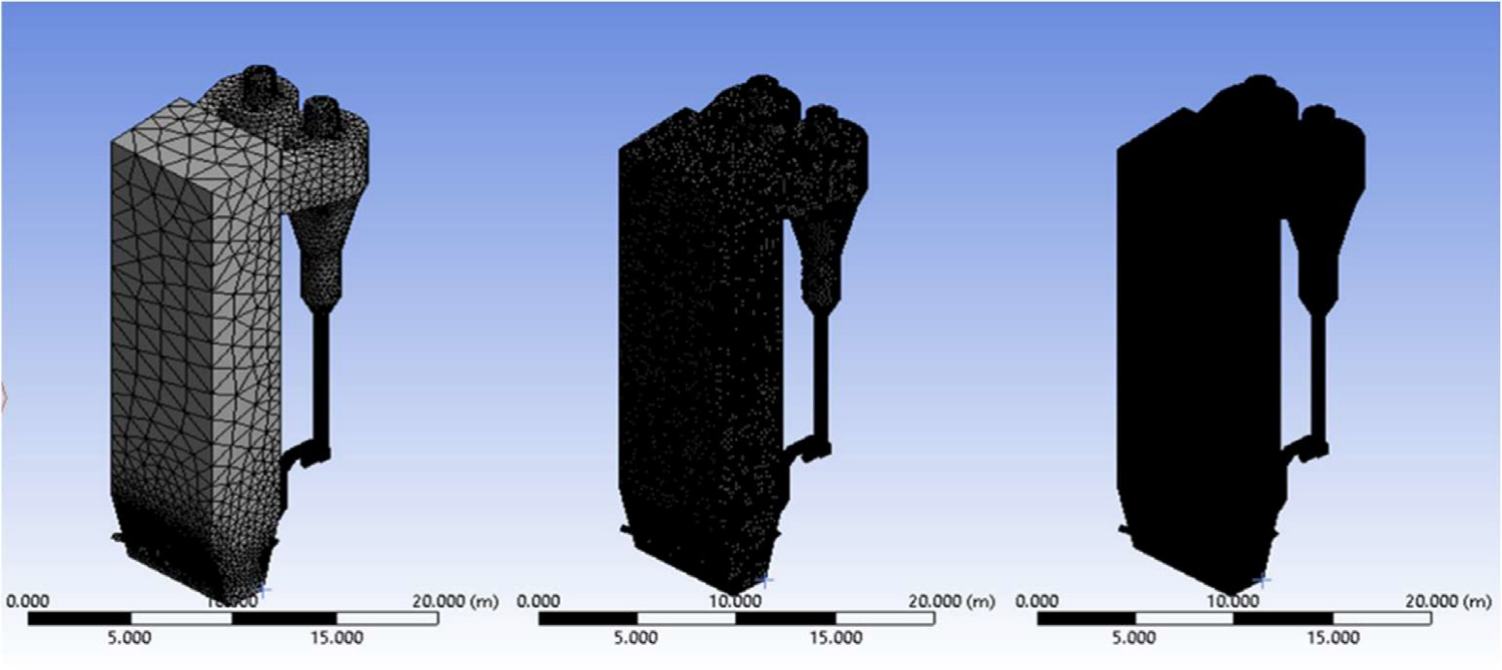
Meshing refinement: (a) Mesh by default (b) 1st modified mesh (c) 2nd modified mesh.

**Fig. 14:**
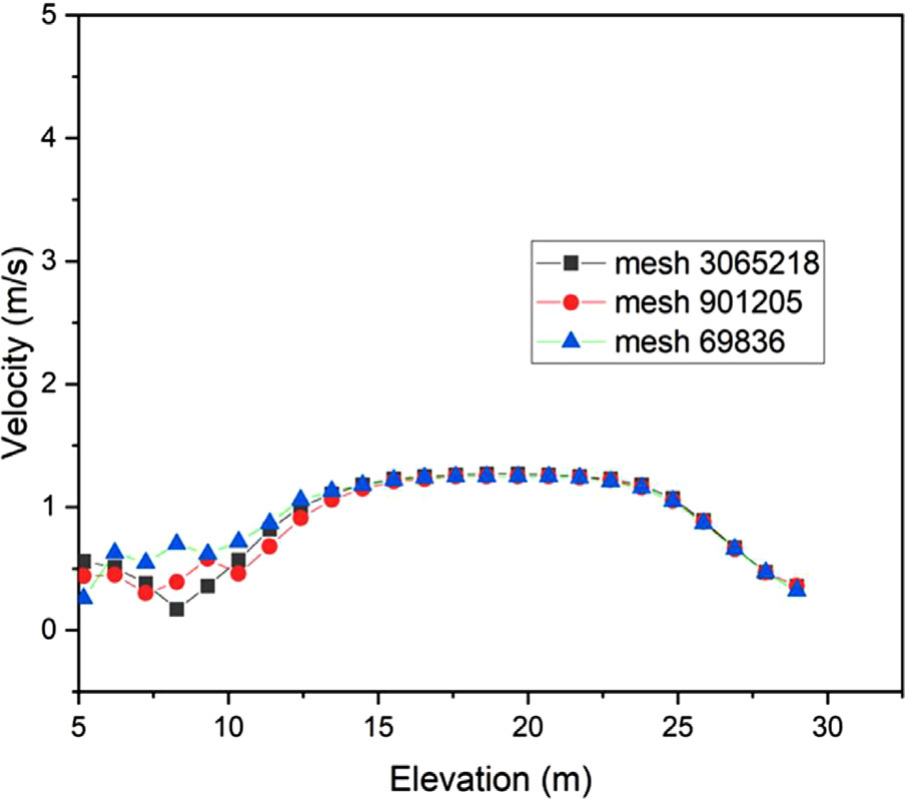
Mesh independence test.

Before conducting the formal calculations, we propose a mesh independence test. In this test, three mesh systems were used, consisting of 69,836 (a), 901,205 (b) and 3,065,218 (c) mesh cells, that can be seen in Fig. 13 using base model (reference model). Fig. 14 illustrates that mesh number of 3,065,218 exhibits a good agreement in terms of velocity along the furnace's central line. We pay attention to velocity distribution since it is a crucial parameter in combustion process, the calculated velocities are plotted along the chamber in the interval of 5–30 m, using 25 measurement points. Based on the stability of velocity vector, we suggest to use a mesh with 3 million elements for the subsequent calculations. This step aims to achieve sufficiently accurate simulation results at an affordable computational cost. It is worth noting that Li et al. [1] have previously conducted successful mesh tests.

**Fig. 15:**
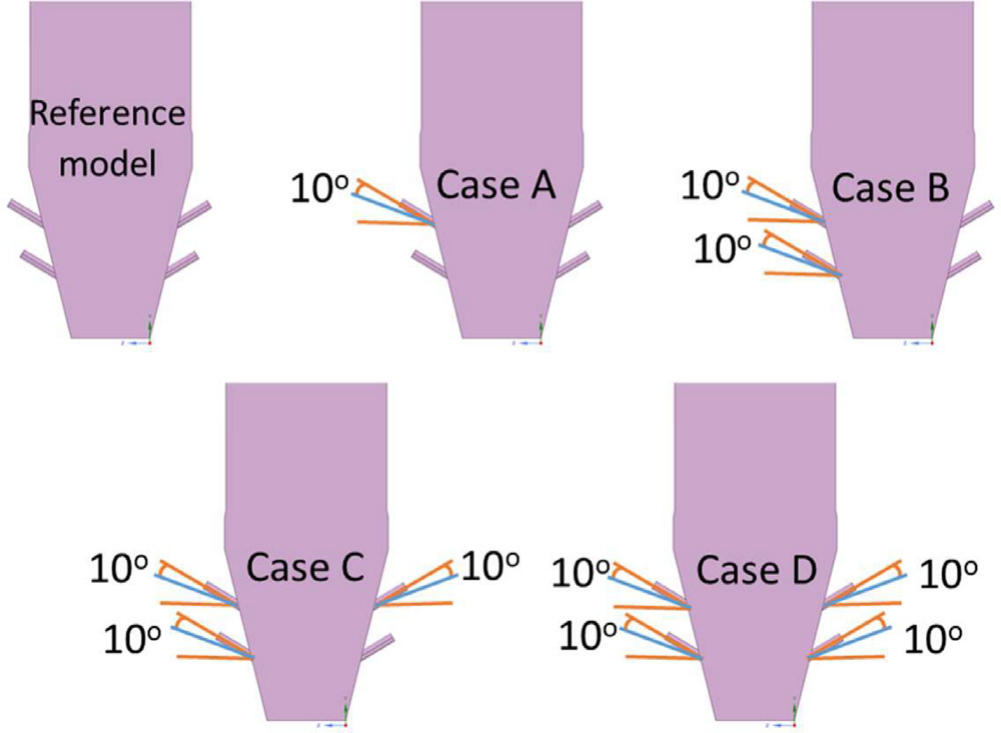
Variation of secondary air inlet direction (angle).

### 3.3 Boundary condition

The boiler was operated at the initial temperature of 600 K and atmospheric pressure. The Semi Implicit Method for Pressure Linked Equation (SIMPLE) [2] was chosen for pressure-velocity coupling problem discretization, and the first-order upwind scheme was used for discretization of momentum, turbulence kinetic energy, and turbulence dissipation rate. Simulations used the transient solver with a maximum of 20 iterations per time step over approximately one hundred time-steps. The flow field was initialized using “Standard Initialization” taking quantities' values at the primary air inlet to initialize the entire domain. Gravity was taken into account by assigning a value of −9.81 m/s in the *y*direction. In total, each case consisted of 2000 iterations.

We used fuel (coal) with specification as can be seen at Table 2. Then, state relation was set into chemical equilibrium and energy treatment is set to non-adiabatic condition. Empirical fuel caloric value is 2.75×10^7 J/kg with operating pressure 101,325 Pascal. The other fluid, air, is set using density of 1.225 kg/m^3^, specific heat (Cp) of 1006.43 J/kg-K, thermal conductivity of 0.0242 W/m-K, viscosity of 1.78×10^-5 kg/m-s, molecular weight of 28.966 kg/kmol. We have modeled combustion reactions via a non-premixed combustion model [3], following the same approach used by Zhu et al. for simulating a pulverized coal boiler [4] . The non-premixed model is applied to solve the coal particles combustion process and account for the coal properties.

Table 4 shows the boundary condition that used in this study. We used constant parameter to determine the effect of angle orientation. Outlet pressure is set into slightly negative as referred into [5]. Then before running each simulation, we set the under relaxation from several parameters. The Solution control under relaxation factor is presented at Table 5.

**Table 4:** Input parameter for boundary condition.

Section	Value
Velocity/temperature of inlet primary air	0.72 m/s 600 K
Velocity/temperature of inlet secondary air	19.96 m/s 600 K
Velocity/temperature of inlet coal	17.84 m/s 300 K
Outlet pressure	-1100 Pa

**Table 5:** The value of under relaxation factor.

Parameter	Value
Pressure	0.3
Density	1
Body forces	1
Momentum	0.7
Turbulent kinetic energy	0.8
Turbulent viscosity	1
Energy	1
Temperature	1
Mean mixture fraction	1
Mixture fraction variance	0.9
Discrete phase source	0.9

**Fig. 16:**
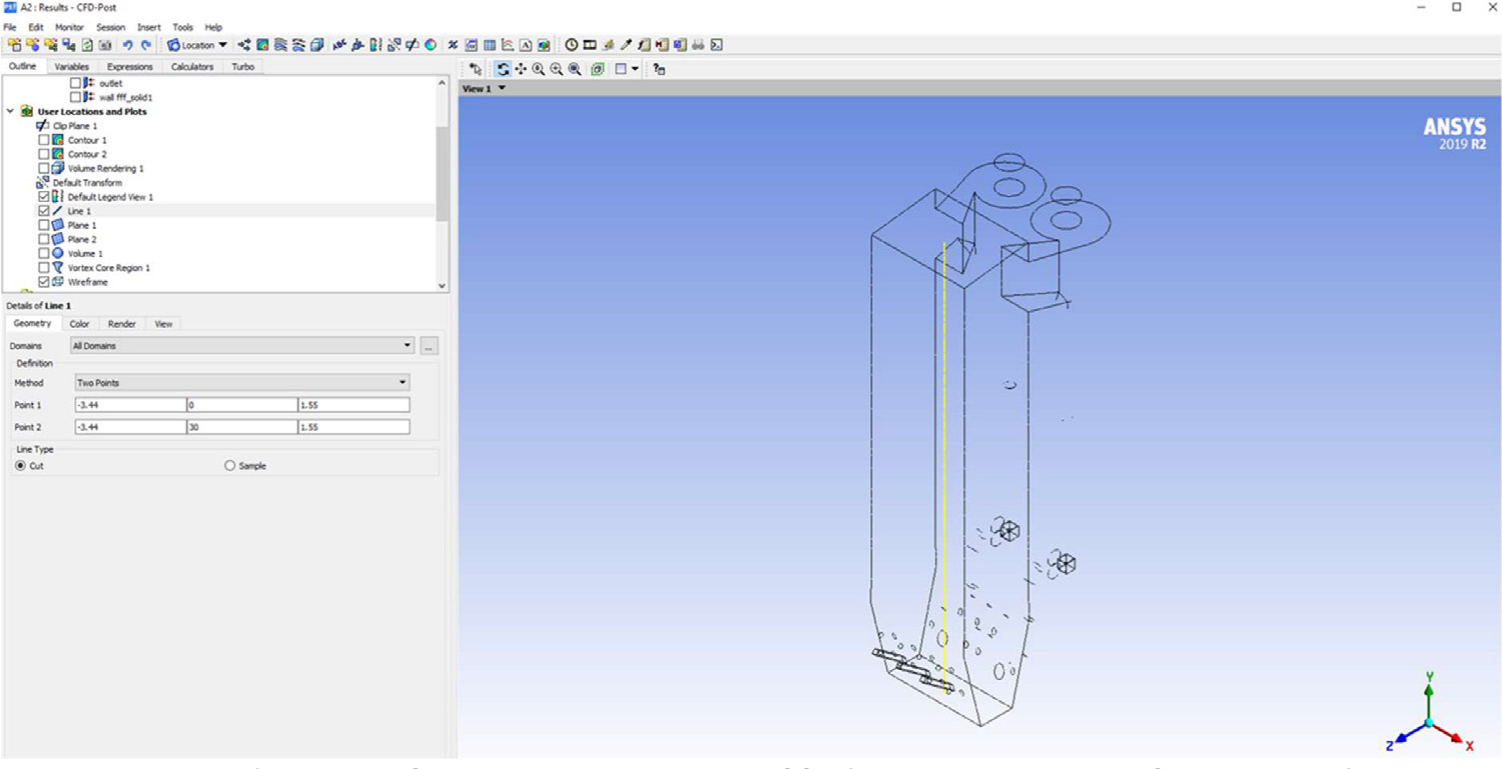
Center line setup for measurement point of fuel, CO_2_, H_2_O, O_2_ mass fraction, total energy, velocity and temperature.

After simulation, we defined a new coordinate system which describes by a yellow line in Fig. 16. This yellow line coincides with the Y axis of the new coordinate. Fig. 16 shows the location of center line of the combustion chamber and coordinates setup. Then we used “insert chart” function and exported of fuel,$\;CO_{2},\;H_{2}O,\;O_{2}$ mass fraction, total energy, velocity and temperature parameter.

## Limitations

None.

## Ethics Statement

The authors have read and follow the ethical requirements for publication in Data in Brief and confirming that the current work does not involve human subjects, animal experiments, or any data collected from social media platforms.

## CRediT authorship contribution statement

**Nurdin Hasananto Teguh:** Writing – original draft. **Lilis Yuliati:** Writing – review & editing. **Eko Siswanto:** Validation, Writing – review & editing. **Djarot B. Darmadi:** Writing – review & editing, Methodology.

## Data Availability

Dataset of CFD simulation modified air angle of CFB Boiler (Original data) (Mendeley Data)
